# From Da Vinci Si to Da Vinci Xi: realistic times in draping and docking the robot

**DOI:** 10.1007/s11701-020-01057-8

**Published:** 2020-02-20

**Authors:** Emma M. van der Schans, Marijn A. J. Hiep, Esther C. J. Consten, Ivo A. M. J. Broeders

**Affiliations:** 1 Department of Surgery, Meander Medical Center, Maatweg 3, Amersfoort, The Netherlands; 2 Faculty of Electrical Engineering, Mathematics and Computer Science, Institute of Technical Medicine, University of Twente, Drienerlolaan 5, Enschede, The Netherlands; 3 Department of Surgery, University Medical Center Groningen, Hanzeplein 1, Groningen, The Netherlands

**Keywords:** Robot-assisted surgery, Docking, Draping, Learning curve, Time efficiency

## Abstract

Robot-assisted surgery is assumed to be time consuming partially due to extra time needed in preparing the robot. The objective of this study was to give realistic times in Da Vinci Xi draping and docking and to analyse the learning curve in the transition from the Si to the Xi in an experienced team. This prospective study was held in a hospital with a high volume of robot-assisted surgery in general surgery, urology and gynaecology. Times from the moment patients entered the operating room until the surgeon took place behind console were precisely recorded during the first 6 weeks after the implementation of the Xi. In total, 65 procedures were performed and documented. The learning curve for the process of draping and docking the robot was reached after 21 and 18 cases, respectively. Mean times after completion of the learning curve were 5 min for draping and 7 min for docking and were statistically different from mean times before completion of the learning curve (*p* values < 0.01). In dedicated teams netto extra time needed for preparing the Xi can even be reduced to just the time needed for docking. Thus, setting up the robot should have limited impact on overall time spent in the operation room.

## Introduction

The use of robotic systems for surgical procedures has increased rapidly over the past 2 decades. Although robot-assisted surgery has several advantages over conventional endoscopic surgery, its costs and prolonged operative times remain a subject of debate. High costs are not only ascribed to high purchase and maintenance costs of the robot, but to longer operating times as well [1–3].

Prolonged overall operating room (OR) times are partially due to extra time needed for preparing the robot (draping the robot, positioning the robot, calibrating and docking the robotic arms) [4–6]. However, focus is mainly put on total operation time and console time [7]. There is a lack of reliable data about extra time needed for setting up the robot.

Although new robotic devices are entering the surgical market, Intuitive Surgical (Sunnyvale, CA, USA) is still the market leader. In 2014, it has launched their fourth generation Da Vinci, the Da Vinci Xi Surgical System. With the recent introduction of this latest model in our experienced robotic centre, we precisely documented times of draping and docking the Xi with the objective to analyse the learning curve of the transition of an experienced robot OR team to a new device and to create reliable data about extra time needed to set up the Da Vinci Xi Surgical System. We hypothesized that the transition from the Si to the Xi would be mastered quickly and that extra time needed for preparing the robot can be kept to a minimum in a dedicated OR team.

## Methods

### Study design

This prospective study was held in a teaching hospital with a high volume of robot-assisted laparoscopic surgery. Since the purchase of a Da Vinci Si early 2011, its assistance has been used for a dedicated set of procedures in general surgery, urology and gynaecology. In March 2019, it was replaced by the Xi system. Set-up times of all consecutive robot-assisted surgeries performed from the start until 6 weeks after implementation of the Xi were documented by one of two independent researchers with no other obligations during the set-up phase.

### Surgical teams

During this study, there were three gastro-intestinal surgeons (GI surgeons), two urologists and two gynaecologists using the robot. Thirteen well-trained scrub nurses, dedicated to robot-assisted surgery for a substantial part of their daily work, completed the teams. Although the scrub nurses rotate between the three specialties, these rotations are kept to a minimum. All team members involved had significant experience with the Si model.

Prior to definite transition of the Si to the Xi, all employees of the robot OR team (consultants and nurses) attended an on-site training with the new model. The training course was held by a representative of Intuitive Surgical. This representative attended the OR during the first surgeries with the Xi to give instructions to the OR team if needed.

### Outcome measurements

**Fig. 1:**
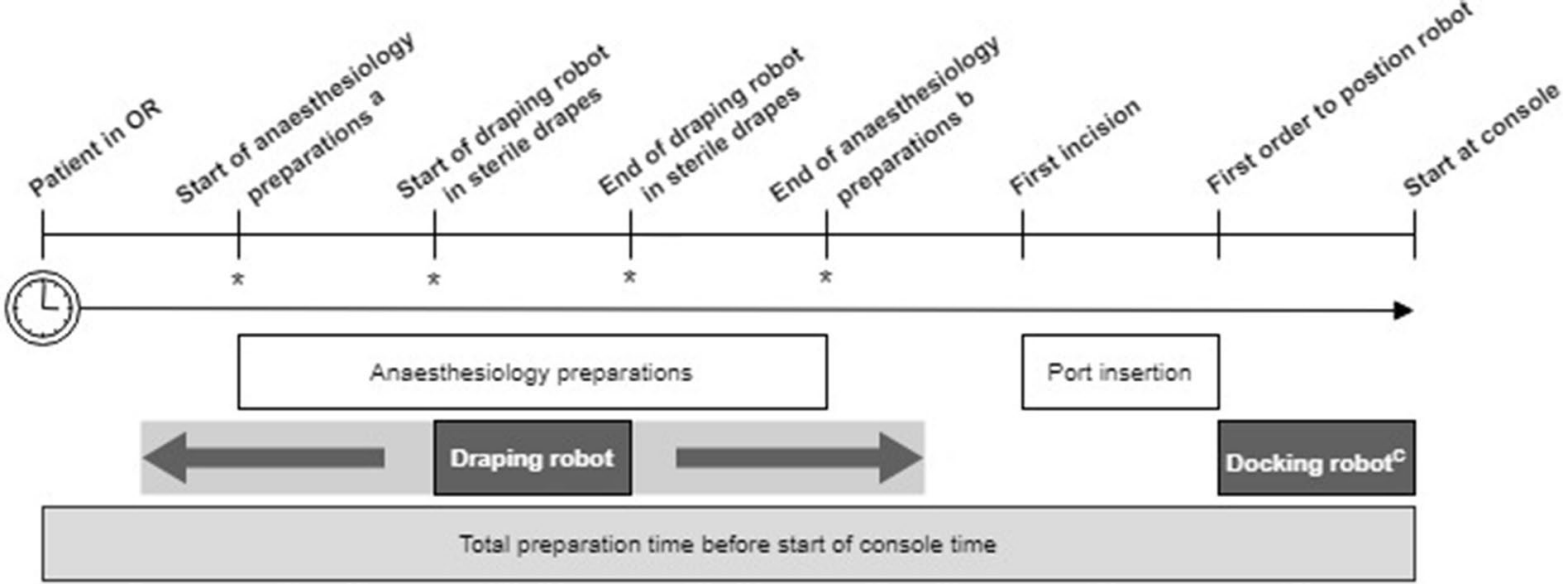
Schematic time sequence illustration of OR preparations. Timeline and timeframes are not scaled. Not all steps in this schematic sequence are in chronological order necessarily. The order of the moments marked with a * can shift between themselves or take place in parallel. Hence, ‘Draping robot’ can be executed completely or partially before, within or after ‘Anaesthesiology preparations’. This depends on OR team efficiency. **a** Start of pre-oxygenation can start any time after the time out has been done. **b** Including putting the patient in correct position for surgery but not sterile draping of the patient. **c** For all procedures the camera arm plus three robotic arms were connected

From the moment a patient entered the OR until the surgeon started at the console, time points of several different steps were recorded. All times were noted in HH:mm:ss. See Fig. 1 for the definitions of each time point and subsequent time frames differentiated. The steps ‘draping robot’ and ‘docking robot’ are seen as dedicated time needed for preparing the robotic device. Draping the robot is a step performed by two scrub nurses. It can start as soon as the patient has been positioned and the bed removed from the OR. Furthermore, draping does not interfere with the preparations done by the anaesthesiologist. This means both activities can be done in parallel. As long as the scrub nurses perform the draping efficiently and start in a timely matter, the total time spent in OR is not adversely affected.

Docking is a step performed by the surgeon, the scrub nurse, and the circulating nurse together (one for positioning and one assisting in calibrating and docking the arms).

### Statistical analysis

Learning curves of robot draping and docking times were analysed using CUSUM analysis. This method enables visualization of trends within the data. It is a frequently used method to assess the process of gaining competence in performing new surgical procedures [8, 9]. CUSUM is the running total of differences between the individual data points and the mean of all cases. First, procedures were ordered chronologically. The CUSUM robot draping for the first procedure was the draping time of the first case minus the mean draping time of all cases; the CUSUM robot draping for the second case was the previous case’s CUSUM added to the difference between draping time of the second procedure and the mean of all cases. This was repeated for each procedure. The same analysis was done for robot docking. The learning phase was defined from the first case to the case representing the inflection point of the CUSUM curve where it changes from a positive to a negative gradient. A stable process is considered as a consistently downward trend or fluctuation around the *x*-axis [9].

Means (with standard deviation) of draping and docking times before and after this point were analysed with independent samples *t* tests. Differences between specialties were assessed by one-way ANOVA. Statistical analysis was performed using SPSS v. 24.0 (IBM Corp., Armonk, NY, USA). A *p* value of < 0.05 was considered significant.

## Results

All 65 robot-assisted operations performed during the first 6 weeks of using the Da Vinci Xi were recorded. The GI surgeons accounted for 26 procedures, the urologist for 25 procedures and the gynaecologists for 14 procedures. Overall mean draping and docking times were 5.6 ± 1.4 and 7.8 ± 2.7 min, respectively (Table 1). Times of draping and docking the robot did not statistically differ between the three surgical disciplines (*p* values 0.366 and 0.207, respectively).

**Table 1:** Robot draping and docking times

Cases	Mean ± SD (minutes)	*P* value*
Robot draping		
1–65	5.6 ± 1.4	
1–21	6.9 ± 1.2	< 0.001
22–65	5.0 ± 1.1	
Robot docking		
1–65	7.8 ± 2.7	
1–18	10.3 ± 3.3	0.001
19–65	6.9 ± 1.6	

*Independent samples *t* test

**Fig. 2:**
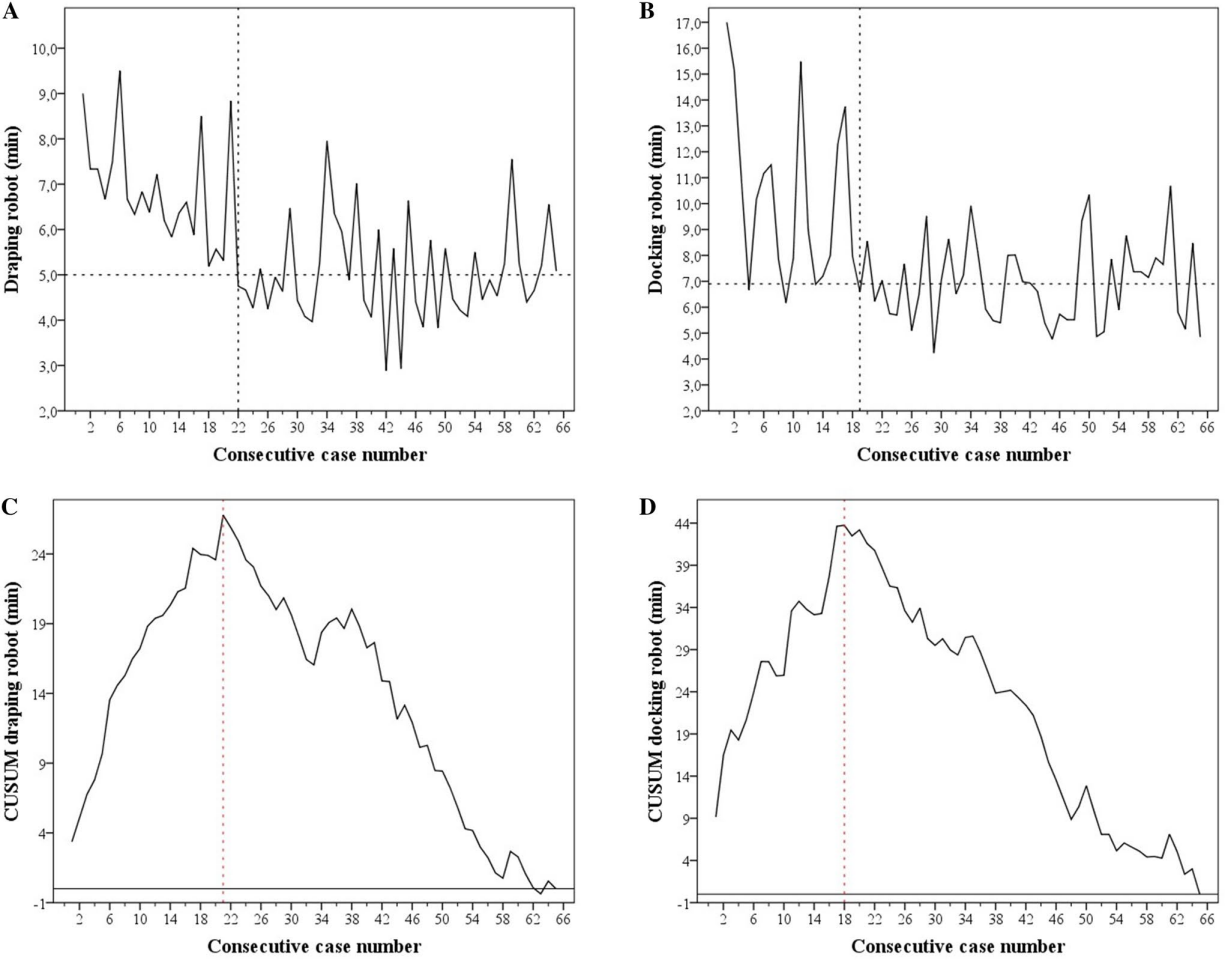
Institutional robot draping and docking times and CUSUM plots. **a**, **b** Represent draping and docking times versus consecutive case number. The vertical dashed lines indicate the first case after completion of the learning phase of the associated step. The dashed horizontal lines indicate the mean time after completion of the learning phase of the associated step. **c**, **d** Represent the plots of CUSUM versus consecutive case number. The vertical dashed lines indicate the inflection point of the CUSUM curves

Times of draping and docking per consecutive procedure are plotted in Fig. 2a, b. CUSUM plots of these steps are depicted in Fig. 2c, d. The learning phase of the whole robot OR team for robot draping and docking was completed after 21 and 18 procedures, respectively. Mean time per step after completion of these learning curves was 5.0 ± 1.2 for draping and 6.9 ± 1.6 min for docking. Mean times before and after completion of the learning curves were statistically significant (*p* value ≤ 0.001; Table 1).

In 36 cases (55.4%), the draping procedure was finished before the anaesthesiology preparations were completed. In 29 cases (44.6%), the draping procedure was still ongoing whilst the anaesthesiology preparations were finished. In all 65 cases, the duration of the anaesthesiology preparation took longer than the duration of robot draping (mean 14.6 ± 4.6 min). A significant difference was seen in number of cases in which the robot draping was completed before the end of anaesthesiology preparations within the three surgical disciplines (*p* = 0.014).

## Discussion

Prolonged operating time remains one of the arguments against robot-assisted surgery. Extra time needed in robot-assisted surgery can be split up in extra time needed for preparing the robot and in actual procedure time. Although literature holds an abundance of data about console times, there is a gap in knowledge about exact times needed in the set-up phase [5–7]. This study is the first to precisely monitor these set-up times in experienced OR teams working with a Da Vinci Xi. It is also the first to provide information on the duration of adapting to a new robotic device.

The presented data show that there is a relatively short period of 18–21 procedures for an institution with an experienced Da Vinci Si staff to adapt to the new Xi system. This transition phase leads to a limited amount of extra OR time. Draping the robot can be achieved in 5.0 min and can be completed within the preparation time needed by the anaesthesiologist. This means that the step of robot draping can be seen as an avoidable delay and does not necessarily affect the overall time spent in the OR. Results show, however, that this is strongly dependant on OR team efficiency. An efficient way of time management was seen in 55% of the observed procedures in this study. A difference was found in frequency in which this most optimal form of time management happened within the OR teams of the different specialties. Attitude within a team could contribute to this.

The step of robot docking always requires extra time when comparing it to conventional laparoscopic procedures. This step can be achieved in 7 min on average. Unlike many other studies, docking time did not include port placement as this step is just as well performed in conventional laparoscopy and should not be considered as extra time [10, 11].

Iranmanesh et al. conducted a similar study from 2006 to 2008 by precisely monitoring draping and docking times of their first experience with the Da Vinci surgical system. In their series of 96 procedures (all general surgery, eight different surgeons, unknown number of scrub nurses), they found median draping times of 22 min (range 9–50) [5]. This is considerably longer then the results found in our institute. Their median docking times were 10 min (range 2–70) which approaches our results more closely. The difference found could be due to the inexperience of their team with robotic surgery at the start of their study and the frequency of robot-assisted surgery (96 procedures in 30 months versus 65 procedures in 6 weeks in our study). Also, the enhancements made in the Xi of simpler docking, laser guided port placement and boom-mounted robotic arms could have played a role in the faster times of draping and docking we found. Comparison to other studies is hard and unreliable due to unclearly described definitions of reported time frames [3, 12–16], different way of docking (three arms or single-port) [6, 17, 18], method of data collection and/or to retrospective study designs [3, 17–19].

Robot-assisted surgery, especially the phase before the surgeon starts behind the console, is a team effort. A limitation of this study is that the composition of the OR teams per specialty was not completely fixed. This was caused by a daily rotating work schedule of our scrub nurses and more than one operating consultant per specialty. However, rotation was mainly kept within one of the surgical disciplines in an effort to minimize the variability. Due to the variable composition of the surgical teams, we were not able to analyse the learning curves per OR team separately. Instead, an institutional learning curve was given. This represents a realistic workflow in a hospital where different specialties use the robotic device. Therefore, it enhances the generalizability of our results to other high-volume practices. Furthermore, the fact that there were no statistically significant differences found in robot draping and docking times between the three specialties supports our method.

In conclusion, both draping and docking times in robotic surgery can be kept to a minimum. CUSUM analysis showed that there is a short learning phase of 21 cases in setting up the robot when a new device is introduced. In dedicated OR teams netto extra time needed for preparing the Xi can be reduced to 7 min needed for docking. Hence, preparing the Da Vinci Xi should have a limited effect on overall time spent in the OR. With a growing number of robot-assisted surgeries, an expending availability of this technology and new manufacturers of surgical robots entering the market, these are valuable data for clinics implementing a (new) robot.
